# Beyond Protein Building Blocks: A Review of Biological Roles and Therapeutic Potential of Free Amino Acids

**DOI:** 10.3390/ijms262311264

**Published:** 2025-11-21

**Authors:** Denitsa Petkova, Savina Stoyanova, Georgi Dinkov, Milen G. Bogdanov

**Affiliations:** 1 Faculty of Chemistry and Pharmacy, Sofia University St. Kliment Ohridski, 1 Jammes Bourchier Blvd., 1164 Sofia, Bulgaria; denitsaip@uni-sofia.bg (D.P.); savinais@uni-sofia.bg (S.S.); 2 IdeaLabs, LLC, 18th Street NW #700, Washington, DC 20036, USA; dinkovg@georgetown.edu

**Keywords:** free amino acids, metabolism, biological activity, biomarkers

## Abstract

While free amino acids (FAAs) are often regarded as simple building blocks for proteins, various studies show they have more complex roles in the body. This review expands on the FAA’s functions, emphasizing their influence on diverse biological processes. It covers their significance in metabolism, energy production, and the synthesis of neurotransmitters, hormones, and antioxidants. FAAs also serve as signaling molecules that regulate critical pathways related to cell growth, autophagy, and metabolic control. The review highlights their impact on the immune system and their essential roles in gut health, nutrient sensing, and metabolic communication. Drawing on recent findings, we emphasize the importance of measuring FAA levels in biological samples and suggest that their supplementation could be beneficial in clinical nutrition, treating metabolic or immune disorders, and preventing sarcopenia. Overall, FAAs are presented as key signaling agents and biomarkers, with potential for targeting their levels to improve health and treat diseases.

## 1. Introduction

Amino acids (AAs) are organic compounds featuring an amino group (-NH_2_) and a carboxyl group (-COOH), which give them their distinctive properties and functions. They form the basic building blocks for protein synthesis and act as precursors in various metabolic pathways. With the exception of glycine, all AAs can exist in both L- and D-isoforms. Most D-AAs, aside from D-arginine, D-cystine, D-histidine, D-lysine, and D-threonine, can be converted back into L-AAs in animals [1].

Based on their role in protein synthesis, AAs can be classified as proteinogenic (PAAs), which indicate their incorporation into proteins during translation, and non-proteinogenic (NPAAs)—those that are not. PAAs can be further subdivided based on their nutritional significance: essential, non-essential, and conditionally essential. Essential amino acids (EAAs) must be obtained from the diet, as the body is unable to synthesize them. These include valine, isoleucine, leucine, methionine, phenylalanine, tryptophan, threonine, histidine, and lysine [1,2]. Non-essential amino acids (NEAAs) are those that the body can synthesize—alanine, aspartic acid, asparagine, glutamic acid, and serine. Furthermore, there are conditionally essential, or semi-essential amino acids (SEAAs), which become essential under specific physiological conditions or stress situations—arginine, cysteine, glycine, taurine, glutamine, proline, and tyrosine [1,2]. The classification of AAs extends beyond mere functional categories; structural and biochemical characteristics also play a vital role. For instance, AAs can be categorized as polar, nonpolar, acidic, and basic. These classifications underscore the significance of AAs properties in determining protein folding and functionality, as well as their interactions with other molecules [3,4].

As is widely recognized, AAs are primarily the fundamental units from which proteins are constructed. The process of protein synthesis encompasses two principal stages: transcription and translation. During transcription, the genetic information stored in DNA is transcribed into messenger RNA (mRNA), which subsequently migrates to the ribosome, the site of translation. AAs are assembled in a precise sequence dictated by the mRNA to form polypeptides, which subsequently fold into functional proteins [5,6]. Research underscores the critical role of AAs in the translation process. Each AA correlates with a specific codon within the mRNA sequence, and transfer RNA (tRNA) molecules are instrumental in translating this information. tRNA molecules transport AAs to the ribosome, where they are incorporated sequentially into the elongating polypeptide chain. This process is subject to tight regulation, and factors affecting AA availability can markedly influence overall rates of protein synthesis. For example, studies have demonstrated that diminished AAs availability hampers muscle protein synthesis, thereby emphasizing the dependence of protein synthesis on AA supply [7,8]. The activation of the mechanistic target of rapamycin (mTOR) pathway constitutes a critical response to AAs availability. This pathway functions as a core regulator of cellular growth, both under normal circumstances and in pathological states such as cancer, as well as in protein synthesis. It integrates signals related to nutrient availability, including AAs, and hormones such as insulin, which facilitate the initiation phase of translation [9,10].

Beyond their fundamental role as protein building blocks, numerous studies have documented additional unique functions of FAAs in the human body (Table A1). These include involvement in signaling and metabolic pathways, energy production, cell growth, autophagy, and the synthesis of neurotransmitters, hormones, and antioxidants. Notably, no comprehensive review has yet summarized these functions. In this article, we systematize the biological roles of FAAs beyond protein synthesis, focusing on their impact on the immune system, gut health, nutrient sensing, metabolic signaling, and other related functions. Our primary objective is to highlight the significance of measuring FAA levels in biological samples for improved detection of specific health conditions related to deficiency or excess of a particular FAA. We also propose their potential use as supplements in clinical nutrition to reduce stress and help address metabolic and immune disorders.

## 2. Review of Individual AAs

### 2.1. Alanine

Alanine is an NEAA with diverse functions within biological systems, notably in metabolism and neurotransmission. One of its principal functions involves its participation in AA metabolism, whereby it is converted into pyruvate and glutamate—energy molecules [11,12]. Additionally, alanine serves as a substrate for gluconeogenesis, particularly during periods of fasting or intense physical activity [12]. Beyond its metabolic roles, alanine also contributes to neurotransmission as a neuromodulator, influencing other neurotransmitters such as glutamate and gamma-aminobutyric acid (GABA) [13,14], and is essential for maintaining synaptic plasticity and overall cerebral function [15,16]. Furthermore, alanine is involved in the synthesis of vital biomolecules, including aspartate, glutamate, and carnosine [17], as well as facilitating nitrogen transport within the organism [18,19].

Although it is not frequently addressed in clinical literature, alanine deficiency may result in various symptoms and conditions, such as dihydropyrimidine dehydrogenase (DPD) deficiency. This genetic disorder impacts pyrimidine metabolism, leading to impaired conversion of uracil to β-alanine [20,21]. Research suggests that alanine deficiency can also disrupt the regulation of β-alanine activation of GABA_A and glycine receptors [22,23] and impair immune responses [24,25]. Furthermore, reduced alanine levels have been linked to metabolic disorders affecting hepatic function and hypoglycemia [26,27].

Conditions associated with elevated alanine are seldom documented; however, increased brain alanine levels have been observed as a result of hyperammonemia [28,29].

### 2.2. Arginine

Arginine, an SEAA, plays a multifaceted role in various biological processes and is crucial for maintaining physiological homeostasis. One of arginine’s most significant functions is its role as a precursor for the synthesis of nitric oxide (NO) [1,30] and facilitation of the detoxification of ammonia by converting it into urea [31,32]. Furthermore, arginine is involved in the synthesis of creatine, glutamate, proline, agmatine, and polyamines [30,33]. It is also essential for muscle growth and repair [34,35], wound healing and tissue repair [36], and embryo implantation and growth [37,38]. Arginine influences the mTOR signaling pathway [39] and participates in the proliferation and differentiation of T cells [40], as well as in lipid metabolism [41,42].

The deficiency of SEAAs may result from inadequate dietary intake, increased metabolic demand, or pathological conditions that affect its synthesis or utilization. Arginine deficiency can impair immune function and lead to cardiovascular complications due to decreased synthesis of NO [43,44]. Moreover, arginine’s role in synthesizing creatine, a vital compound for energy metabolism in the brain, emphasizes its significance in preserving cognitive function [45]. Additional manifestations of arginine deficiency include impaired wound healing [36], as well as conditions associated with elevated arginase activity, such as liver disease and certain inflammatory states [46,47]. Conversely, arginase deficiency can lead to the accumulation of ammonia and arginine [47,48]. Arginine depletion has been recognized as a potential therapeutic strategy for cancer [49,50].

Excessive arginine intake may result in heightened NO production through the activation of inducible nitric oxide synthase (iNOS), which has been associated with inflammatory responses and tissue damage [51,52]. Paradoxically, although NO plays a crucial role in the immune response against foreign pathogens such as viruses and bacteria, chronically elevated NO levels can induce systemic immunodeficiency owing to NO’s toxicity to all cell types when present in sufficiently high concentrations. One of the early hypotheses regarding the progression of HIV infection to AIDS involved elevated NO levels attributable to recreational drug use involving nitrate-containing substances that act as precursors for NO [53]. Furthermore, increased NO levels are recognized as a trigger for the reactivation of latent viral infections, such as herpes, and patients with herpes infections are routinely advised to avoid arginine-rich foods [54]. Elevated arginine levels may also cause metabolic imbalances affecting protein synthesis [55,56] and can lead to increased production of compounds classified as uremic toxins [57,58].

### 2.3. Asparagine

Asparagine is an NEAA possessing numerous functions. It is critical for optimal protein synthesis, particularly under conditions of nutrient deprivation [59], and is essential for cellular adaptation to metabolic stress, such as regulating cellular responses to glutamine depletion [60]. It plays a vital role in T cell activation and function [61,62] and mediates important signaling pathways, including mTOR signaling [63,64]. Additionally, it can influence tumor growth and progression [63,65].

The deficiency of asparagine is frequently associated with genetic disorders, particularly mutations in the asparagine synthetase (ASNS) gene. Such mutations result in severe neurological impairments, which can be attributed to asparagine depletion in the brain or the accumulation of aspartate and glutamate, leading to increased neuronal excitability and damage [66,67]. Reduced levels of asparagine also adversely affect immune function [61]. Finally, asparagine deficiency has been correlated with diminished growth and survival of cancer cells [65,68].

There is limited information regarding the specific conditions and symptoms associated with asparagine excess. Elevated levels of asparagine may potentially contribute to increased tumor growth and survival [65,68]. A high ratio of asparagine to aspartate has been correlated with an increased risk of type 2 diabetes with advancing age [69].

### 2.4. Aspartic Acid

Aspartic acid is an NEAA existing in two enantiomeric forms: L- and D-aspartic acid. The L-form is mainly involved in protein synthesis, while the D-form is important for neurotransmission and hormonal regulation [70,71]. It plays a key role in neurotransmission as an excitatory neurotransmitter in the central nervous system (CNS), influencing cognitive functions like learning and memory by activating the N-methyl-D-aspartate (NMDA) receptor [70,72]. Aspartic acid also serves as a precursor for synthesizing other AAs and neurotransmitters such as asparagine, arginine, nucleotides, and D-aspartic acid [1,71]. D-Aspartate significantly impacts the endocrine system, regulating the release of various hormones [72,73].

While aspartic acid is synthesized within the human body, its concentrations may be affected by dietary intake and metabolic conditions. Deficient levels of aspartic acid can result in asparagine deficiency and neurological symptoms. Furthermore, such a deficiency may contribute to the accumulation of ammonia due to its role in the urea cycle, which is particularly pertinent in conditions such as hepatic disease [71,74]. Low concentrations of aspartic acid may also impair the synthesis of other AAs and nucleotides [1,71]. An inadequate level of D-aspartic acid has the potential to lead to hormonal imbalance, thereby negatively impacting the reproductive system [73,75].

One of the primary concerns regarding aspartic acid excess is its potential impact on neurotransmission, as excitotoxicity—neuronal damage and cell death due to overstimulation of receptors [76,77]—and hormonal imbalances [78,79].

### 2.5. Cysteine

Cysteine is an SEAA characterized by its thiol (-SH) group. Its primary functions include stabilizing protein structures through disulfide bonds, which enable proteins to preserve their native conformations under various environmental stresses [80,81]. Cysteine residues within proteins serve as critical sites for redox reactions, acting as sensors and regulators of oxidative stress [82,83]. The role of cysteine in antioxidant mechanisms is further demonstrated through its participation in the synthesis of glutathione (GSH), a prominent intracellular antioxidant [84,85]. Additionally, cysteine’s capacity to bind metal ions such as zinc and iron is essential for enzymatic catalysis and signal transduction pathways [82,86]. Furthermore, cysteine is involved in the biosynthesis of key biomolecules, including coenzyme A, taurine, and H_2_S [87,88].

A deficiency of cysteine can significantly impact human health. The primary concerns associated with inadequate levels of this AA include heightened susceptibility to oxidative damage, compromised immune function [89,90] and cognitive decline, which may increase the risk of neurodegenerative diseases such as Alzheimer’s and Parkinson’s [90,91]. Insufficient cysteine levels can also result in deficiencies of coenzyme A and taurine [87,92], as well as contributing to the development of Type 2 diabetes, particularly within the context of oxidative stress [92,93].

Excessive levels of cysteine can exert toxic effects by inhibiting essential metabolic pathways, particularly those involved in energy production, protein metabolism, insulin resistance, and diabetes [94,95]. Elevated cysteine concentrations may induce cellular toxicity through disruption of iron homeostasis via an ROS-mediated mechanism [96]. Increased plasma cysteine levels have been observed in individuals with cardiovascular disease (CVD) compared to healthy controls [97,98]. High cellular levels of GSH are characteristic of cancer [99] and numerous studies have demonstrated that GSH depletion [100] or its oxidation into the disulfide form GSSG exerts therapeutic effects across various tumor types [101]. Ultimately, the GSH/GSSG ratio serves as one of the principal “redox” indicators, reflecting the delicate balance between oxidation and reduction within metabolic reaction chains [102]. This ratio is comparable to other significant redox ratios such as NAD^+^/NADH, acetoacetate/beta-hydroxybutyrate, and pyruvate/lactate.

### 2.6. Glutamic Acid

Glutamic acid, also referred to as glutamate, is an NEAA acid that fulfills significant biological functions, particularly within the CNS and various metabolic pathways. As the most predominant excitatory neurotransmitter in the brain, glutamate is instrumental in numerous CNS activities, including synaptic transmission, neuronal communication, learning, and memory formation [103,104]. Moreover, it functions as a precursor and nitrogen donor in the synthesis of other AAs, purines, and pyrimidines [105]. The balance between glutamate and GABA, a metabolite derived from glutamate, is vital for maintaining the equilibrium between excitatory and inhibitory neurotransmission within the brain [106,107]. Additionally, glutamate is a constituent of the tricarboxylic acid (TCA) cycle [108]. It also plays a role in regulating insulin secretion, being capable of stimulating insulin release from pancreatic beta cells [109,110]. Finally, glutamic acid contributes to the synthesis of GSH [85,111].

A deficiency of glutamic acid is not a common issue in human health. This deficiency can potentially lead to cognitive deficits and difficulties in learning and memory, owing to glutamate’s role as a neurotransmitter [112,113]. Since glutamic acid is the precursor for GABA, insufficient glutamate levels may subsequently contribute to decreased GABA levels and neurological disturbances [106,107]. Certain disorders, including depression and anxiety, have also been associated with lower levels of glutamic acid [114,115].

Excess glutamic acid in the human body can lead to a variety of conditions and symptoms, primarily due to its role in the CNS. It has been associated with excitotoxicity, which can result in the influx of calcium ions (Ca^2+^) into neurons, potentially causing cell death and abnormal electrical activity in the brain. Such conditions may manifest as seizure disorders [116,117], schizophrenia, and major depressive disorder [118,119]. Additionally, acute neurological events such as stroke and traumatic brain injury [117,120] as well as ischemic stroke [121,122], are linked to elevated glutamate levels. Elevated glutamate can also induce retinal excitotoxicity, leading to cell death and contributing to diseases such as glaucoma [123]. NMDA receptor, for which glutamate functions as a ligand, represents a significant pharmacological target for numerous health conditions. Pharmacological agents that act as antagonists to this receptor have demonstrated a broad spectrum of benefits, particularly regarding neurological and mental health disorders [124].

### 2.7. Glutamine

Glutamine is an SEAA that plays a multifaceted role in various biological processes. It serves as an important carbon and nitrogen donor for biosynthesis [125,126] and is regarded as a conditionally EAA due to its support of lymphocyte and macrophage proliferation and activity during inflammatory conditions [127,128]. Its functions include the regulation of pH balance via metabolization to ammonia in the kidneys [129,130]. Additionally, glutamine is a precursor for the synthesis of neurotransmitters glutamate and GABA [131] and participates in GSH synthesis, a major antioxidant [132,133]. It also influences the mTOR signaling pathway [134]. Furthermore, glutamine is often described as a “fuel” for tumor growth, given that cancer cells exhibit modified metabolic pathways involving glutaminolysis [135,136].

Glutamine deficiency can arise from various conditions and can lead to a range of symptoms that significantly impact health. One of the most severe forms of this deficiency is congenital glutamine synthetase (GS) deficiency, a rare genetic disorder characterized by a lack of the enzyme responsible for synthesizing glutamine, which causes severe neonatal encephalopathy and is associated with chronic hyperammonemia [137]. Insufficient levels of glutamine can also lead to impaired immune responses, increased susceptibility to infections, delayed wound healing [133,138], sepsis [139,140], cognitive deficits [141,142], and intestinal dysfunction [143,144]. Moreover, glutamine deficiency may contribute to the exacerbation of certain conditions, such as cancer, where tumor cells frequently exhibit “glutamine addiction” [145,146]. This dependence on glutamine is believed to play a significant role in cancer cachexia, due to the protein wasting of healthy tissues that occurs in an effort to supply glutamine for the rapidly proliferating cancer cells [147].

Excessive levels of glutamine are less frequently addressed than deficiencies and may result from high dietary intake, supplementation, or metabolic dysregulation. Such excess is linked to hyperammonemia—reducing the necessity for ammonia conversion into glutamine within the brain and liver [148,149]—as well as tumor progression and resistance to therapeutic interventions [150,151].

### 2.8. Glycine

Glycine has the simplest structure and is also SEAA, particularly important as a neurotransmitter in the CNS, where it assumes a dual role: as an inhibitory neurotransmitter, crucial for the modulation of synaptic transmission and the regulation of motor and sensory pathways within the spinal cord and brainstem [152,153], and as an excitatory neurotransmitter—serving as a co-agonist with glutamate at NMDA receptors [153,154]. Additionally, glycine facilitates the survival of neural stem cells (NSCs) during embryonic development [155,156], influences the migration of microglial cells, which are essential for immune responses within the CNS [157,158], and exhibits cytoprotective properties across various non-neuronal cells, including immune cells [159,160]. Furthermore, this AA participates in the biosynthesis of numerous biomolecules, such as GSH, creatine, purines, collagen, and heme [161,162].

Glycine deficiency is relatively uncommon; however, reduced levels of glycine have been associated with conditions such as obesity, type 2 diabetes, non-alcoholic fatty liver disease (NAFLD) [163,164], and impaired hemoglobinization of erythroid cells, which may potentially lead to anemia [162]. Insufficient glycine may also contribute to defective collagen synthesis and weakening of connective tissue [165]. One of the most notable aspects of glycine is its capacity to extend both average and maximum lifespan when incorporated into the diet at concentrations of 1–2% of the diet weight. The effects observed from dietary glycine supplementation appear to emulate the benefits associated with caloric restriction, implying that it is not solely caloric intake but rather the composition of the diet that influences lifespan [166].

One of the most notable conditions associated with elevated glycine levels is non-ketotic hyperglycinemia (NKH), a genetic disorder characterized by the accumulation of glycine in the blood and cerebrospinal fluid (CSF) due to a deficiency in the glycine cleavage system [167]. Other manifestations of glycine excess include sensorimotor gating disturbances, particularly in the context of psychiatric disorders such as schizophrenia [168,169] and metabolic dysregulation [161].

### 2.9. Histidine

Histidine is an EAA that influences various physiological processes and metabolic pathways. One of its primary biological functions is serving as a precursor in the synthesis of histamine, which modulates numerous physiological functions, including gastric acid secretion, immune responses, and neurotransmission [170,171]. Additionally, histidine acts as a buffer within the physiological pH range due to its imidazole side chain [172] and participates in the coordination of metal ions, such as zinc and copper, in diverse enzymatic processes and cellular signaling pathways [172,173]. The antioxidant capacity of histidine and its derivatives is vital in mitigating oxidative damage caused by reactive oxygen species (ROS) [172,173]. Furthermore, histidine plays a role in metabolic regulation by enhancing insulin sensitivity and decreasing markers of systemic inflammation [174,175].

Histidine deficiency can result in various physiological and psychological conditions. One of the most significant consequences of a deficiency of histidine is its effect on mental health, presenting symptoms associated with major depressive disorder [176,177]. Additional conditions linked to inadequate histidine levels encompass impaired intestinal health, reduced antioxidant capacity [178], as well as dermatological and joint issues [172,179].

Excessive levels of histidine in the body can lead to several adverse health conditions and symptoms. One of the primary concerns associated with histidine excess is the potential for histidinemia, a metabolic disorder characterized by elevated levels of histidine in the blood with neurological symptoms [180]. In addition to neurological effects, excessive histidine can also lead to gastrointestinal disturbances, a decrease in appetite, taste, and smell [181,182], and increased zinc excretion, potentially resulting in zinc deficiency in some individuals [175]. Elevated levels of histidine may also result in histamine excess, which has a proven role in allergies and chronic inflammation, driving processes such as cancer and CVD. Histamine receptor antagonists have been shown to provide numerous health benefits not only for allergies, but also for gastro intestinal tract conditions, cancer, and neurological diseases [183].

### 2.10. Isoleucine

Isoleucine is an EAA, one of the three branched-chain amino acids (BCAAs). A primary function of isoleucine is its involvement in muscle protein synthesis, as it stimulates the mTOR signaling pathway, which is essential for muscle growth and repair [184,185]. Isoleucine exerts a positive influence on insulin sensitivity and glucose metabolism, thereby promoting metabolic health [186,187]. It is classified as both a ketogenic and glucogenic AA [188]. Furthermore, isoleucine can enhance immune function [189,190].

Isoleucine deficiency can lead to various clinical conditions and symptoms, primarily due to its vital role in protein synthesis. One of the most notable conditions associated with isoleucine deficiency is acrodermatitis dysmetabolica, observed in patients with Maple Syrup Urine Disease (MSUD) [191,192]. Additionally, isoleucine deficiency has been linked to muscle wasting and diminished physical performance [193,194]. Reduced levels of isoleucine have also been reported in conditions such as major depressive disorder [177,195].

Excessive levels of isoleucine may induce a spectrum of physiological and metabolic disturbances, including heightened insulin resistance [196,197]. Furthermore, an overabundance of isoleucine exerts adverse effects on growth and development in specific animal models, leading to growth retardation, which is likely attributable to the accumulation of toxic metabolites resulting from isoleucine oxidation [198,199]. Elevated concentrations of this AA have been associated with increased adipose tissue deposition in certain animal models, implying an alteration in lipid metabolism [194,200].

### 2.11. Leucine

Leucine is an EAA with a branched-chain structure, participating in a variety of biological functions, particularly in protein metabolism, cellular signaling, and energy homeostasis. A primary function of leucine is its role as a key regulator of the mammalian target of rapamycin complex 1 (mTORC1), which is essential for controlling protein synthesis and cellular growth in response to nutrient status [201,202]. Moreover, leucine functions as a regulator of energy metabolism and insulin sensitivity [203], as well as a modulator of feeding behavior and energy balance within the CNS [204,205]. Additional roles of leucine include regulation of autophagy, a process vital for maintaining cellular homeostasis and responding to stress [201,206], muscle preservation [207,208], influences on cardiovascular health [209,210], as well as participation in lipogenesis and lipolysis [211,212].

As an EAA, leucine must be obtained through the diet, and its absence can disrupt numerous biological processes. One of the primary concerns associated with its deficiency is impaired protein synthesis, particularly in muscle tissue [201,202]. Insufficient levels of leucine may lead to a disrupted balance of BCAAs [213]. Leucine deficiency has also been associated with cognitive impairments and mood disorders [177,195]. Nonetheless, leucine-restricted diets have demonstrated the potential to reduce fat mass, improve glucose regulation, and enhance hepatic insulin signaling [213,214].

Excess leucine, although less frequently discussed than deficiency, can result in various health problems and metabolic disruptions. Among the most prominent conditions linked to leucine excess is MSUD, a hereditary disorder characterized by the impaired metabolism of BCAAs, primarily manifesting through neurotoxicity, with symptoms including seizures, developmental delays, and cognitive impairments [215,216]. In addition to MSUD, elevated levels of leucine may contribute to imbalances in AA levels, such as deficiencies in isoleucine and valine [217,218]. Furthermore, an excess of leucine has been demonstrated to compete with tryptophan for transport into the brain, thereby leading to decreased serotonin synthesis [219,220].

### 2.12. Lysine

Lysine, classified as an EAA, is pivotal in various biological processes. Beyond its role in protein synthesis, lysine is integral to post-translational modifications (PTMs), particularly acetylation and succinylation, which are essential for regulating protein function, stability, and interactions within the cell [221,222]. Lysine also participates in the biosynthesis of carnitine [223,224]. Additionally, this EAA contributes to malonylation and isobutyrylation in protein modifications [225,226], mediates epigenetic regulation through acetylation [227,228], influences the regulation of hormones such as ghrelin and leptin which are involved in appetite and energy homeostasis [229,230], and is implicated in collagen and elastin cross-linking [231]. Furthermore, lysine plays a significant role in promoting bone health and supporting immune function [232,233].

One of the most notable conditions associated with lysine deficiency is growth and developmental problems, which may be exacerbated by the potential onset of hypoproteinemia [234,235]. Insufficient lysine levels can impair immune responses [236,237] and may lead to the accumulation of lipids [238,239]. Regarding neurological health, lysine deficiency may induce stress, mood swings, and sleep disturbances [240,241]. Lysine is believed to function as an antagonist to arginine, either by decreasing arginine levels or by reducing its conversion to NO. Consequently, lysine supplementation has been employed in viral conditions associated with excess NO, such as the reactivation of latent herpes virus infection [242].

Lysine, although indispensable for numerous physiological processes, can induce adverse effects when consumed in excess. Elevated lysine levels may precipitate oxidative stress due to the generation of ROS [243,244], may impose renal stress, and potentially contribute to the development of renal diseases [245], and are associated with gastrointestinal disturbances [246].

### 2.13. Methionine

Methionine is an EAA acid with numerous critical roles within biological systems. Its primary functions include the regulation of gene expression and the stabilization of proteins. It is the initial AA incorporated into newly forming polypeptide chains during translation, serving as the N-terminal residue in most [247]. Additional significant functions of methionine encompass its role as a precursor to S-adenosylmethionine (SAM), a vital methyl donor involved in various methylation reactions [248,249]. It also participates in antioxidative processes through the methionine sulfoxide oxidation-reduction cycle [250,251], regulates cellular signaling pathways essential for cell growth and protein synthesis [252,253], and contributes to the synthesis of other sulfur-containing compounds such as cysteine and subsequently GSH, a primary antioxidant within the body [254,255]. Furthermore, methionine serves as a precursor to polyamines, succinyl-CoA, homocysteine, creatine, and carnitine [256].

Methionine deficiency can induce a range of physiological and metabolic alterations. A principal consequence of methionine deficiency is the modulation of miRNAs and gene expression [257,258]. Dietary restriction of methionine has been demonstrated to enhance insulin sensitivity and promote lipid oxidation in metabolic conditions such as obesity [259,260], as well as to improve cardiovascular health despite the potential risk of hyperhomocysteinemia [258,261], and to augment antioxidative and anti-inflammatory responses [262]. Diets with restricted methionine intake have also been associated with beneficial effects on cognitive functions [263,264] and the suppression of tumor proliferation [265,266]. Additionally, methionine restriction, akin to glycine supplementation, has been shown to emulate caloric restriction effects, thereby extending both the average and maximum lifespan in various animal models [267].

Methionine, although essential for various biological functions, can become detrimental when present in excessive amounts, particularly within the context of dietary consumption. One of the most significant consequences of methionine excess, especially when combined with B vitamins and folate deficiency, is hyperhomocysteinemia—the elevation of homocysteine levels in the bloodstream. This condition is a well-established risk factor for CVD, including atherosclerosis and thrombosis [268,269]. Furthermore, elevated levels of methionine and homocysteine can induce neurotoxicity, cognitive impairment, and inflammation [269,270]. High concentrations of methionine may also contribute to renal impairment [271,272] and oxidative stress, characterized by increased production of ROS [273,274]. Additionally, excess methionine, similar to cysteine and tryptophan, has been shown to directly suppress thyroid function [275], which may elucidate why restricting methionine intake in the diet yields numerous positive effects on weight management, diabetes, cancer, and lifespan.

### 2.14. Phenylalanine

Phenylalanine constitutes an essential aromatic AA with significant biological roles, primarily through its metabolism and transformation into other biologically important compounds. A principal function of phenylalanine is its conversion into tyrosine and subsequently dopamine, one of the major neurotransmitters [276,277]. Additionally, phenylalanine serves as a precursor for the biosynthesis of melanin [278], and it may influence the secretion of gut hormones [279,280].

Phenylalanine deficiency, though less frequently addressed than its excess, can result in a variety of significant health concerns. One of the primary conditions associated with phenylalanine deficiency is impaired growth and development, particularly in infants and children with Tyrosinemia type 1 [281,282]. Additional manifestations may encompass cognitive impairments and neurocognitive issues, as well as dermatological conditions such as eczema and other skin disorders [281].

Excess phenylalanine, particularly in cases of phenylketonuria (PKU), induces a spectrum of severe health complications and symptoms that can substantially impair an individual’s quality of life. PKU is characterized as an autosomal recessive disorder distinguished by a deficiency of the enzyme phenylalanine hydroxylase (PAH), which catalyzes the conversion of phenylalanine to tyrosine [283]. A primary and severe consequence of phenylalanine accumulation is neurotoxicity, as elevated levels interfere with neurotransmitter synthesis by competing with other large neutral AAs for transport across the blood–brain barrier [284,285]. This competitive inhibition may also contribute to increased anxiety and mood fluctuations [286]. Additional manifestations of PKU include dermatological issues and hypopigmentation [287,288]. Furthermore, elevated phenylalanine concentrations have been correlated with cardiovascular complications [289,290].

### 2.15. Proline

Proline, an SEAA, fulfills vital biological functions across a diverse range of organisms, from plants to mammals. A primary biological role of proline is its involvement in osmotic regulation, where it functions as an osmolyte [291,292]. Proline possesses significant antioxidant properties, effectively scavenging ROS and safeguarding cells from oxidative damage, which is particularly important during exposure to heavy metals or hydrogen peroxide [293]. Additionally, proline, in conjunction with glycine, constitutes a crucial component of collagen, contributing to its structural stability through the stabilization of the triple helix configuration [294,295], and is essential in protein folding owing to its distinctive cyclic structure [296,297]. The oxidative metabolism of proline results in the formation of glutamate and the subsequent release of energy [297]. This metabolic pathway also supplies energy to cancer cells and plays a role in their metabolic reprogramming [298,299]. Furthermore, the proline-P5C cycle is instrumental in maintaining redox homeostasis within cells, especially under conditions of oxidative stress or metabolic challenges [300,301]. It is also recognized that proline influences neurotransmission [302,303].

A deficiency of proline in plants may result in a diminished capacity to respond effectively to osmotic stress [292,304]. In human physiology, proline deficiency is less frequently examined. The role of proline as an antioxidant suggests that inadequate proline levels could contribute to heightened oxidative stress. Moreover, reduced proline levels may impair collagen synthesis, thereby compromising the integrity of connective tissues [305,306].

It has been observed that elevated proline content can alter the ultrastructure of essential organelles such as chloroplasts and mitochondria, and the generation of ROS [307]. In humans, excessive proline is principally associated with hyperprolinemia, which manifests symptoms including neurological impairment and psychiatric disorders, notably schizophrenia [303,308]. Prolonged administration of excess proline may also induce oxidative damage [309,310].

### 2.16. Serine

Serine, an NEAA, performs numerous critical biological functions. A primary role involves its participation in one-carbon metabolism, which is vital for the synthesis of nucleotides, SAM, and NADPH [311,312]. Furthermore, serine serves as a precursor for other AAs, including glycine and cysteine [313]. It is also indispensable for lipid metabolism, being necessary for the biosynthesis of phosphatidylserine and sphingolipids [314,315]. Additional roles encompass regulation of immune responses [316,317], detoxification, and antioxidant defense via its involvement in GSH biosynthesis [318]. Moreover, serine is essential for neuronal development, neurotransmitter synthesis, and overall brain function [319,320]. It plays a significant role in the proliferation and survival of cancer cells [321,322]. D-serine functions as a co-agonist of NMDA receptors and is involved in neuronal activity, including synaptic plasticity, neurodevelopment, learning, and memory processes [323,324]. Additionally, serine is pivotal in the biosynthesis of androgens and estrogens, as serine phosphorylation of cytochrome b5 is essential for the activity of the 17,20-lyase enzyme, which constitutes the rate-limiting step in the production of these steroids [325]. Deficiency of D-serine can manifest as cognitive impairments and neurodevelopmental disorders [326,327].

Serine deficiency is associated with a range of clinical conditions and symptoms, primarily affecting neurological function and development. The principal indicators of serine deficiency disorders include congenital microcephaly, seizures, and severe psychomotor delay [319,320]. It is established that serine deficiency can lead to developmental delays and neurological impairments from birth, with some cases identifiable even prenatally [319,328]. Additionally, serine deficiency may be linked to diminished antioxidant defenses and an increased susceptibility to oxidative damage [329].

High levels of L-serine can lead to the accumulation of D-serine [330]. Elevated levels of D-serine may result in a range of toxicological effects and physiological disturbances, chiefly impacting the CNS and renal function. Excessive D-serine interferes with normal neuromuscular operations and development [323], and it can also provoke nephrotoxic effects, culminating in glucosuria and polyuria [331,332].

### 2.17. Threonine

Threonine is an EAA that plays a fundamental role in various metabolic pathways and physiological functions. It is particularly significant in the synthesis of mucins, which are glycoproteins constituting a major component of mucous membranes, thereby being crucial for the preservation of mucosal integrity and immune function [333,334]. Threonine additionally acts as a precursor for the biosynthesis of other biomolecules, including AAs such as glycine, serine, and isoleucine [335,336], and is indispensable in the PTMs of proteins, especially phosphorylation [337,338]. Its catabolism is vital for the maintenance of pluripotent and multipotent stem cells in both plant and animal systems [339,340].

A primary condition associated with threonine deficiency is compromised intestinal function [341,342], alongside adverse effects on metabolic processes, particularly those pertaining to energy metabolism, such as glucose absorption capacity [333,343]. Inadequate levels of threonine may result in a weakened immune response, rendering individuals more vulnerable to infections and diseases [333,344]. Additional conditions linked to threonine deficiency include decreased body weight gain and impaired growth rates [345,346], as well as disruptions in the estrous cycle, which may indicate potential impacts on fertility and reproductive health [347].

Threonine, although vital for various physiological functions, may cause adverse effects when present in excessive amounts. A primary concern associated with threonine overabundance is its potential to induce metabolic disturbances, such as the accumulation of toxic intermediates within metabolic pathways, including homoserine [348]. Elevated levels of threonine may also pose negative neurological impacts [349]. Furthermore, excess threonine can result in diminished protein synthesis and growth [346,350].

### 2.18. Tryptophan

Tryptophan is an essential aromatic AA with diverse functions in human physiology. It acts as a precursor for significant biomolecules such as serotonin and melatonin, which are crucial for neurotransmission, mood regulation, and circadian rhythm management [351,352]. Furthermore, tryptophan influences the immune system through the production of kynurenines, which can impact immune responses and inflammatory processes [352,353]. It is also a precursor for the synthesis of NAD [352]. This AA affects gastrointestinal health and overall well-being [354,355], and plays a role in sleep regulation via its conversion into melatonin [356].

Tryptophan deficiency impacts multiple health domains, including neuropsychiatric, metabolic, and immune systems. A significant consequence of this deficiency is its effect on serotonin levels, which are associated with mood disorders such as depression and anxiety [357,358]. Insufficient tryptophan intake can impair cognitive functions, including memory and learning [358,359], disrupt energy metabolism, and result in fatigue and muscle weakness [360]. Additionally, it may increase susceptibility to infections and inflammatory processes [357,361]. Notably, certain research suggests that brain tryptophan depletion, which decreases serotonin levels, might exert anti-fatigue effects, thereby supporting the central fatigue hypothesis [362].

Excessive consumption of tryptophan may result in a variety of medical conditions and symptoms that could negatively impact health. A principal concern associated with high levels of tryptophan is its potential to disturb serotonin concentrations [363,364]. Although serotonin plays a crucial role in mood regulation, an overabundance may cause serotonin toxicity [365]. Additionally, excess tryptophan can lead to increased production of kynurenine and its derivatives, which may contribute to oxidative stress and inflammatory responses [366,367].

### 2.19. Tyrosine

Tyrosine is a semi-essential aromatic AA that plays various roles within biological systems. One of its most vital functions is the synthesis of catecholamines, including dopamine, norepinephrine, and epinephrine, which are essential for numerous physiological processes such as mood regulation, stress response, and cardiovascular functioning [368,369]. Additionally, tyrosine serves as a precursor for thyroid hormones and melanin [370,371]. Furthermore, PTMs such as tyrosine phosphorylation, nitrosation, and sulfonation are crucial for cellular signaling and regulatory mechanisms [372,373].

The effects of low tyrosine levels can manifest in various ways, particularly impacting neurological function. A primary condition associated with tyrosine metabolism is tyrosine hydroxylase (TH) deficiency, an autosomal recessive disorder characterized by a significant reduction in the production of dopamine, norepinephrine, and epinephrine [374,375]. Overall health may also be compromised by tyrosine deficiency, which can lead to depression and anxiety due to decreased levels of neurotransmitters such as dopamine and norepinephrine [376,377].

One primary concern associated with excess tyrosine is its potential impact on neurotransmitter synthesis, which may lead to increased physiological arousal and symptoms of anxiety, overstimulation of the CNS, and cognitive impairments [378,379]. There is also accumulating evidences indicating that elevated tyrosine levels are correlated with obesity and insulin resistance [380,381]. Additionally, high tyrosine levels can result from a rare genetic disorder known as tyrosinemia. Tyrosinemia type I can cause severe liver dysfunction, neurological crises, and rickets due to the accumulation of toxic metabolites [382]. Tyrosinemia type II may lead to skin lesions, corneal deposits, and neurological impairments [383,384], while tyrosinemia type III is associated with neurological symptoms and intellectual disability [385,386].

### 2.20. Valine

Valine is an essential BCAA. Valine, along with leucine and isoleucine, are main components of muscle protein [184], which highlights its significance in muscle maintenance and repair. Other important functions of the AA involve stimulation of the mitochondrial function in muscle cells, which is essential for energy production and overall metabolic health [387,388], influence on insulin sensitivity [389,390], influence on triglyceride synthesis and overall lipid profiles in various animal models [388,391]. Valine can also enhance intestinal health and immune status [392,393] and can indirectly be involved in regulation of neurotransmitter synthesis [394].

Valine deficiency can lead to a range of physiological and metabolic disturbances, given its essential role in various biological processes. Lower levels of valine can contribute to increased insulin sensitivity [213,389], but valine deficiency has been shown to result in weight loss due to decreased protein synthesis and stimulated protein and fat breakdown [193,395]. Additionally, it may have neurotoxic effects [396,397].

While valine is essential for numerous biological functions, its excessive presence may result in toxic effects. Elevated levels of this AA can impair insulin signaling and glucose metabolism, thereby contributing to the development of type 2 diabetes [398,399] and cardiovascular diseases CVD [398,400]. Furthermore, high concentrations of valine may disrupt lipid metabolism and promote NAFLD [401,402].

## 3. Daily AA Intake Requirements

To provide a comprehensive overview of daily AA requirements, it is essential to address that each AA’s specific requirement varies depending on factors such as age, gender, activity level, and physiological conditions. According to established sources, the total daily requirements for EAAs have been thoroughly documented. The WHO/FAO/UNU guidelines indicate that adults require specific amounts of each EAA: histidine (10 mg/kg), isoleucine (20 mg/kg), leucine (39 mg/kg), lysine (30 mg/kg), methionine (15 mg/kg), phenylalanine (25 mg/kg), threonine (15 mg/kg), tryptophan (4 mg/kg), and valine (26 mg/kg) [403,404].

Protein intake, which is the primary source of AAs, is vital for the maintenance of various physiological functions. Regular assessments of dietary protein intake reveal significant implications for health and disease prevention. For adults, a common recommendation is approximately 0.83 g of protein per kilogram of body weight. This intake level aligns well with maintaining overall bodily functions, muscle synthesis, and metabolic activities [405].

In athletes and individuals engaged in regular physical activity, the protein requirement can significantly increase. Recommendations suggest that the intake of protein, preferably with higher BCAA content, should be around 1.2 to 2.0 g per kilogram of body weight per day, depending on the type, intensity, and duration of the training [406,407].

Vegetarian and vegan diets also necessitate careful consideration regarding AA intake. A comprehensive evaluation has suggested that while these diets can meet AA requirements, they often require more strategic combinations of food sources to ensure adequate EAA intake, particularly lysine and methionine, usually found in high concentrations within animal proteins [408,409].

Requirements for children are usually higher because of their more rapid growth and development. Research indicates that the requirements for protein intake typically vary from 1.0 to 1.5 g per kilogram of body weight per day in children [405,410]. Additionally, studies suggest that children consuming plant-based diets may struggle to meet their AA needs effectively due to lower digestibility and limitations from most plant proteins [411,412] compared to protein of animal origin (e.g., eggs, dairy, meat, etc.).

Another age group with higher protein intake requirements comprises the elderly. Aging presents unique challenges for protein metabolism, necessitating a higher intake of specific AAs to counteract muscle loss and inefficiencies in protein synthesis [413,414]. Current guidelines suggest that elderly individuals should consume at least 1.0–1.2 g/kg of protein per day, particularly due to decreased anabolic responses to dietary proteins [415,416]. The individual AA requirements are notably higher for the elderly as well; essential AAs such as leucine play a significant role in stimulating muscle protein synthesis [417,418].

## 4. Nutritional Sources of AAs

Animal-based protein sources are traditionally recognized for their high protein content and complete AA profiles. For example, meat, poultry, eggs, and dairy products such as milk and cheese are known to be rich in protein and contain all the EAAs required by the human body [419,420]. Animal-based proteins are especially favored by athletes [421]. Fish is also a significant source of high-quality protein and EAAs. Research indicates that fish not only boasts a high protein content but also contains a favorable AA composition rich in taurine, lysine and methionine [422].

In the realm of vegetarian sources, legumes, nuts, seeds, and certain grains emerge as viable protein-rich alternatives. For instance, pulses such as lentils and beans are lauded for both their high protein content and beneficial AA profiles [420,423]. Proteins from vegetable sources do not have adequate amounts of all nine EAAs, usually lacking one or two. That is why people who exclude animal proteins from their diets should consume a variety of plant-based proteins to ensure consumption of all EEAs [420,424].

Mushrooms are increasingly recognized for their nutritional value, particularly as a source of protein. Research indicates that edible mushrooms can contain protein levels comparable to some legumes. Certain varieties have been reported to exhibit protein content ranging from approximately 25% to 50% on a dry weight basis [425,426]. For example, Agaricus bisporus (common button mushroom) has been identified to contain significant amounts of AAs like glutamic acid and aspartic acid, which are vital for muscle metabolism and overall bodily functions [427,428].

In terms of AA composition, protein quality assessments reveal that animal proteins are generally acknowledged for their higher quality compared to many plant-derived proteins. It is important to accurately evaluate protein quality based on AA digestibility and composition, particularly concerning EAAs [429]. To optimize protein intake and meet physiological needs, it is relevant to structure meal plans that combine high-protein foods from both animal and plant sources.

## 5. Possible Therapeutic Effects of AA Blends

When assessing the potential therapeutic effects of AAs, it is essential to consider that their metabolic pathways are significantly interconnected. A substantial body of existing research explores the interactions between various AAs, their shared transport mechanisms, and their capacity to enhance or inhibit specific functions. However, numerous recently identified tendencies necessitate further investigation. Additionally, another critical area of research pertains to the effects of different AA blends, particularly EAA blends, on various physiological processes, as AAs may act synergistically to enhance metabolic functions or exhibit antagonistic effects that could impede particular pathways.

The inclusion of a higher EAA intake in the diet has been demonstrated to provide significant benefits across various physiological contexts, such as enhancing muscle strength and mass, and improving body composition, particularly when combined with resistance training [430,431]. Moreover, the rate at which AAs are delivered to tissues after exercise can influence recovery effectiveness. Clinical research has also shown that supplementing with EAAs can enhance muscle preservation in people undergoing calorie restriction, weight loss, or conditions that compromise muscle health [432,433]. EAA supplementation has been linked to improved kidney function in adults and prevention of liver damage [434,435,436], reduced inflammation [437,438,439] and decreased risks of dyslipidemia via modulation of the lysine/arginine ratio [440,441]. Research also indicates that a higher intake of EAAs, especially a greater proportion of leucine, is necessary to sufficiently stimulate MPS, particularly in elderly individuals [442]. A very recent and interesting study demonstrated that leucine is important not only for structural integrity of the organism (e.g., building muscle and bones) but also for physiological function. Namely, leucine was found to be uniquely responsible for maintaining mitochondrial function, modulate autophagy and helps cells adjust respiration in relation to changing environmental and nutritional conditions [443].

The question of what form EAA should be additionally introduced in the diet is also quite important. The ratio of EAA/NEAA in proteins has been identified to be at most 1:1 (good quality protein), but many protein sources do not reach that ratio in regard to EAA [444,445,446]. Recent studies have documented health benefits from a ratio higher in EAA, suggesting that maintaining the ratio > 1 is critical for promoting healthy growth, supporting metabolic functions, and ensuring nutritional adequacy [447]. An attempt to increase EAA intake through dietary sources of protein would subsequently increase the intake of NEAA, which can either have no additional effects or might actually lead to potential metabolic inefficiencies and result in a bigger pool of nitrogen catabolites, which are known to have a harsh effect on the kidneys and liver. Studies also indicate that an improper ratio of EAA/NEAA and high intake of certain NEAAs may inhibit the absorption or utilization of EAA, disrupting normal physiological processes [448]. Nevertheless, an adequate level of NEAAs must be maintained to support EAA availability. Furthermore, the quantities of individual EAAs should be meticulously assessed, depending on the purpose or necessity of their inclusion. This makes the incorporation of EAAs in supplement form a more judicious approach, as it allows precise formulation of the EAA composition and quantities, while also ensuring their easy bioavailability. As previously mentioned, the levels of methionine and tryptophan naturally present in various foods as part of the EAA profile have been identified as excessive for optimal health. Specifically, reductions in the absolute intake—and consequently as a percentage of total EAA intake—of methionine and/or tryptophan have been associated with numerous beneficial effects, including improved body composition (such as weight loss), enhanced metabolic status (notably insulin resistance), decreased cancer development and progression, and ultimately increased lifespan. Therefore, when formulating EAA mixtures for human supplementation in a laboratory setting, it may be advantageous to alter the ratios of various EAAs significantly from those naturally occurring in most food sources, particularly concerning methionine, tryptophan, and even histidine (the exclusive precursor of the major inflammatory mediator, histamine). When considering the supplementation of EAAs, BCAAs—leucine, isoleucine, and valine—are of most interest due to their unique benefits in muscle protein synthesis, recovery, and overall metabolic health. However, the amounts in which they are taken should be evaluated, because excessive concentration of BCAA can lower dopamine synthesis due to reduced tyrosine uptake in the brain [449], or contribute to adverse cardiovascular outcomes [450]. Another thing to keep in mind is that not all EAA are required to be additionally supplied. Tryptophan, methionine and histidine are already circulating in optimal, even excess concentrations and there is no need for further supplementation. Furthermore, given the recent shift in scientific opinion in regard to clarifying glycine, taurine and beta-alanine as conditionally essential, a more optimal mix of EAA may include six of the original EAA minus the ones mentioned immediately above, while also adding glycine and taurine, usually in a 1:1 ratio (glycine/taurine). Overall, the supplementation of EAAs comes with a couple of challenges, particularly concerning optimal AAs composition, dosage, timing, and specific populations’ responsiveness.

## 6. Conclusions

The significance of FAAs extends far beyond their role in protein synthesis. Given that plasma and serum FAAs are integral components of an intricate metabolic network involving hepatic, muscular, and adipose tissues, their concentrations can serve as indicators of organ-specific dysfunctions and overall metabolic health. Indeed, plasma FAA profiles have been recognized as sensitive characteristics of systemic metabolic imbalance and emerge as promising biomarkers for a range of pathological conditions. Numerous studies corroborate this, establishing relationships with conditions such as CVD, diabetes mellitus, obesity, renal disorders, carcinomas, and thyroid abnormalities. The liquid chromatography-tandem mass spectrometry (LC-MS/MS) is the benchmark method for profiling many AAs simultaneously. However, data quality and comparability across labs depend heavily on standardized protocols, careful control of pre-analytical variables and targeted AA panels to ensure dependable results. For clinical use, future studies should focus on validating methods across diverse populations, standardizing assay procedures, combining data with other omics and clinical information, and exploring the underlying mechanisms to clarify causality and find potential therapeutic targets.

Besides focusing on precise measurement of FAAs, it is also important to explore other ways the field can progress. With the rapid development of artificial intelligence (AI), we believe the future of researching optimal AA intake will shift from broad protein recommendations to a more adaptable, personalized nutritional approach driven by AI. This change involves more than just measuring FAAs; it aims to understand the specific functional and conditional needs of all AAs and their metabolites across different life stages, health conditions, and individual genetic and metabolic profiles. Factors such as a person’s genome, gut microbiome, health status, and circadian rhythms create a complex, multi-variable challenge that is best tackled with AI solutions. By utilizing large datasets from multi-omics studies, wearable devices, and medical records, AI can create predictive models of individual metabolism, discover new biomarkers, and deliver personalized dietary advice in real-time. This synergy between advanced science and AI will facilitate the implementation of precise clinical and athletic nutrition strategies, fostering the development of sustainable, targeted foods. Ultimately, this paradigm shift will transform AA intake from a general dietary guideline into a highly tailored tool for enhancing human health and performance.

## Data Availability

No new data were created or analyzed in this study. Data sharing is not applicable to this article.

## Appendix A

**Table A1:** FAAs discussed in this review, their main biological functions, and documented effects related to deficiency or excess.

Amino Acid	Biological Function	Deficiency	Excess
Alanine non-essential	Conversion into pyruvate and glutamate [11,12] Substrate for gluconeogenesis, particularly during fasting or intense exercise [12] Neuromodulator of glutamate and GABA [13,14] Maintaining synaptic plasticity and overall brain function [15,16] Synthesis of important biomolecules (aspartate, glutamate and carnosine) [17] Transporting nitrogen waste from muscles to the liver [18,19]	Dihydropyrimidine dehydrogenase deficiency (genetic disorder) [20,21] Disruption in the balance of β-alanine activation of GABA_A and glycine receptors [22,23] Metabolic disorders that affect liver function [26,27] Impaired immune responses [24,25]	Increased levels of alanine aminotransferase, a marker for liver damage or dysfunction [28,29] Hyperammonemia [28,29]
Arginine semi-essential	Precursor to NO [1,30] Detoxification of ammonia [31,32] Synthesis of creatine, glutamate, proline, agmatine and polyamines [30,33] Muscle growth and repair [34,35] Influence on the mTOR signaling pathway [39] Embryo implantation and growth [37,38] Proliferation and differentiation of T cells [40] Wound healing and tissue repair [36]	Impaired immune function [43,44] Cardiovascular complications [43,44] Neurological symptoms [45] Impaired wound healing [36] Conditions associated with increased arginase activity, such as liver disease and certain inflammatory states [46,47] Potential therapeutic strategy for cancer [49,50]	Metabolic imbalances [55,56] Excessive NO production [51,52] Increased production of guanidino compounds [57,58]
Asparagine non-essential	Optimal protein synthesis [59] Cellular adaptation to metabolic stress [60] Mediates important signaling pathways (mTOR) [63,64] Influence on tumor growth and progression [64,65] T cell activation and function [61,62]	Asparagine synthetase deficiency [66,67] Neurological impairments [66,67] Impact on the immune function [61] Hinder cancer cell growth and survival [65,68]	Enhanced tumor growth and survival [65,68] Risk of type 2 diabetes with age [69]
L-Aspartic acid non-essential	Synthesis of other AAs and neurotransmitters (glutamate and asparagine) [1,71]	Asparagine deficiency and neurological symptoms Impaired metabolic pathways [1,71] Accumulation of ammonia [71,74]	Excitotoxicity—neuronal damage and cell death due to overstimulation of receptors [76,77]
D-Aspartic acid non-essential	Excitatory neurotransmitter in the CNS [70,72] Neurotransmission and hormonal regulation [70,71]	Hormonal imbalance [73,75]	Hormonal imbalances [78,79]
Cysteine semi-essential	Stabilization of protein structures [80,81] Sites for redox reactions [82,83] Synthesis of GSH [84,85] Enzymatic catalysis and signal transduction pathways [82,86] Synthesis of other essential biomolecules, including coenzyme A and taurine [87,88]	Increased susceptibility to oxidative damage [89,90] Impaired immune function [89,90] Deficiency of coenzyme A and taurine [87,92] Type 2 diabetes, particularly in the context of oxidative stress [92,93] Cognitive decline and increased risk for diseases like Alzheimer’s and Parkinson’s [90,91]	Toxicity due to inhibiting key metabolic pathways, insulin resistance and diabetes [94,95] Cellular toxicity by disrupting the iron homeostasis [96] CVD [97,98]
Glutamic acid non-essential	Primary excitatory neurotransmitter in the CNS [103,104] Nitrogen donor and precursor to other AAs and purines and pyrimidines [105] Part of the TCA cycle [108] Regulation of insulin secretion [109,110] Synthesis of GSH [85,111]	Cognitive deficits, difficulties in learning and memory [112,113] Reduced GABA synthesis [106,107] Neurological disorders, including depression and anxiety [114,115]	Excitotoxicity that can lead to cell death [116] Stroke and traumatic brain injury [117,120] Ischemic stroke [121,122] Abnormal electrical activity in the brain, resulting in seizure disorders [116,117] Schizophrenia and major depressive disorder [118,119] Retinal excitotoxicity [123]
Glutamine semi-essential	Important carbon and nitrogen donor for biosynthesis [125,126] “Fuel” for tumor growth [135,136] Supports the proliferation and activity of lymphocytes and macrophages [127,128] Regulation of pH balance [129,130] Influences on mTOR [134] Precursor to glutamate, GABA, GSH [131]	Congenital glutamine synthetase (GS) deficiency [137] Impaired immune responses [133] Increased susceptibility to infections [133] Delayed wound healing [138] Sepsis [139,140] Cognitive deficits [141,142] Exacerbation in conditions like cancer [145,146] Intestinal dysfunction [143,144]	Hyperammonemia [148,149] Tumor progression and resistance to therapy [150,151]
Glycine semi-essential	Inhibitory neurotransmission—modulation of synaptic transmission [152,153] Excitatory neurotransmission co-agonist at NMDA receptors [153,154] Promotes the survival of NSCs [155,156] Influence the migration of microglial cells [157,158] Cytoprotective properties in various non-neuronal cells, including immune cells [159,160] Synthesis of various biomolecules, including GSH, creatine, purine, collagen and heme [161,162]	Impaired collagen synthesis and connective tissue weakness [165] Obesity, type 2 diabetes [163] NAFLD [164] Impaired hemoglobinization of erythroid cells, potentially resulting in anemia [162]	NKH [167] Sensorimotor gating, particularly in the context of psychiatric disorders such as schizophrenia [168,169] Metabolic dysregulation [161]
Histidine essential	Precursor for synthesis of histamine [170,171] Buffer in physiological pH range [172] Coordination of metal ions, such as zinc and copper, in various enzymatic processes and cellular signaling pathways [172,173] Mitigating damage caused by ROS [172,173] Improves insulin sensitivity and reduces markers of systemic inflammation [174,175]	Symptoms associated with major depressive disorder [176,177] Impaired intestinal health and antioxidant capacity [178] Skin and joint problems [172,179]	Histidinemia, leading to neurological symptoms [180] Gastrointestinal disturbances [181] Decrease in appetite, taste and smell [182] Increased zinc excretion [175]
Isoleucine essential branched-chain	Muscle protein synthesis—stimulates the mTOR signaling pathway [184,185] Both a ketogenic and glucogenic [188] Favorable influence on insulin sensitivity and glucose metabolism [186,187] Enhances immune function [189,190]	Acrodermatitis dysmetabolica, observed in patients with MSUD [191,192] Muscle wasting and impaired physical performance [193,194] Major depressive disorder [177,195]	Induce metabolic dysregulation and increased insulin resistance [196,197] Adverse effect on growth and development [198,199] Increased fat deposition [194,200]
Leucine essential branched-chain	Key regulator of the mTORC1 signaling pathway [201,202] Regulator of energy metabolism and insulin sensitivity [203] Modulator of feeding behavior and energy balance in the CNS [204,205] Autophagy regulation [201,206] Muscle preservation [207,208] Promoting cardiovascular health [209,210] Influences on lipogenesis and lipolysis [211,212]	Impaired protein synthesis, particularly in muscle tissue [201,202] Elevated levels of other BCAAs [213] Cognitive impairments and mood disorders [177,195] Reduced fat mass and improved glucose regulation, and enhanced hepatic insulin signaling [213,214]	MSUD [215,216] Neurotoxicity, particularly in the context of MSUD [215,216] Disturbances in AA balance [217,218] Disrupted serotonin synthesis [219,220]
Lysine essential	Integral to PTMs, particularly acetylation and succinylation [221,222] Involved in the biosynthesis of carnitine [223,224] Epigenetic regulation through acetylation [227,228] Malonylation and isobutyrylation in protein modifications [225,226] Role in regulation of hormones such as ghrelin and leptin [229,230] Collagen and elastin cross-linking [231] Role in bone health and immune function [232,233]	Growth and developmental issues further exacerbated by potential development of hypoproteinemia [234,235] Impaired immune responses [236,237] Potential accumulation of lipids [238,239] Mood instability, sleep disturbances, and cognitive impairments [240,241]	Oxidative stress because of formation of ROS [243,244] Renal stress and potential development of kidney-related issues [245] Gastrointestinal disturbances [246]
Methionine essential	Regulation of gene expression and protein stability [247] Precursor to SAM [248,249] Important antioxidant through the methionine—methionine sulfoxide oxidation-reduction cycle [250,251] Regulation of cellular signaling pathways, crucial for cell growth and protein synthesis [252,253] Conversion to other sulfur-containing compounds like cysteine and consequently GSH [254,255] Precursor to polyamines, succinyl-CoA, homocysteine, creatine, and carnitine [256]	Alternation of miRNAs and gene expression [257,258] Improved insulin sensitivity and promoted fat oxidation [259,260] Improve cardiovascular health, despite potential for hyperhomocysteinemia [258,261] Improved antioxidation and anti-inflammatory response [262] Beneficial effect on cognitive function [263,264] Reduce tumor growth and survival [265,266]	Hyperhomocysteinemia—elevated homocysteine is a well-established risk factor for CVD [268,269] Stimulate inflammatory pathways [269,270] Neurotoxic effects [269,270] Renal impairment [271,272] Oxidative stress—increased production of ROS [273,274]
Phenylalanine essential aromatic	Conversion into tyrosine and subsequently—neurotransmitter synthesis [276,277] Biosynthesis of melanin [278] Impact on gut hormone secretion [279,280]	Impaired growth and development, particularly in infants and children with Tyrosinemia type 1 [281,282] Cognitive impairments and neurocognitive problems [281] Dermatological issues like eczema and other skin problems [281]	PKU impaired conversion of phenylalanine to tyrosine [283] Neurotoxic effect [284,285] Increased levels of anxiety and behavioral problems [286] Skin problems and hypopigmentation [287,288] Cardiovascular complications [289,290]
Proline semi-essential	Osmolyte [291,292] Antioxidant—scavenging ROS [293] Critical component of collagen [294,295] Essential in the folding of proteins due to its unique cyclic structure [296,297] Production of glutamate and a subsequent release of energy, which can also provide cancer cells energy [297,298,299] Maintenance of redox homeostasis through the proline-P5C cycle [300,301] Influence on neurotransmission [302,303]	Oxidative stress Impaired collagen synthesis, leading to compromised connective tissue integrity [305,306] Impaired ability to respond to osmotic stress in plants [292,304]	Hyperprolinemia, associated with symptoms such as neurological impairment and psychiatric disorders, including schizophrenia [303,308] Oxidative stress [309,310] Ultrastructure of essential organelles such as chloroplasts and mitochondria [307]
L-Serine non-essential D-Serine non-essential	Involvement in one-carbon metabolism—synthesis of nucleotides, SAM and NADPH [311,312] Precursor to glycine, cysteine, GSH [313] Important in growth and survival of cancer cells [321,322] Integral to lipid metabolism Regulation of immune responses [314,315] Neuronal development, neurotransmitter synthesis, and overall brain function [319,320] Co-agonist of NMDA receptors [323,324]	Congenital microcephaly, seizures, and severe psychomotor retardation [319,320] Developmental delays and neurological symptoms from birth [319,328] Impaired antioxidant defenses and increased susceptibility to oxidative damage [329] Cognitive impairments and neurodevelopmental disorders [326,327]	Increased D-Serine synthesis [330] Disruptions in normal neuromuscular function and development [323] Nephrotoxic effects, leading to glucosuria and polyuria [331,332]
Threonine essential	Formation of mucins [333,334] Precursor for the synthesis of various biomolecules (glycine, serine and isoleucine) [335,336] Plays a significant role in PTMs of proteins, particularly phosphorylation [337,338] Maintenance of pluripotent and multipotent stem cells in both plants and animals [339,340]	Impaired gut function [341,342] Negative effect on metabolic processes, particularly those related to energy metabolism [333,343] Weakened immune response [333,344] Reduced body weight gain and impaired growth rates [345,346] Disruption in the estrous cycle [347]	Accumulation of toxic intermediates in metabolic pathways, such as homoserine [348] Negative neurological impact [349] Reduced protein synthesis and growth [346,350]
Tryptophan essential aromatic	Precursor to serotonin [351,352] Immune system modulation—production of kynurenines [352,353] Influence on the gastrointestinal function and overall health [354,355] Regulation of sleep and circadian rhythms through its conversion to melatonin [356] Precursor for the synthesis of NAD [352]	Impact on serotonin levels, leading to depression and anxiety [357,358] Deficits in cognitive functions, such as memory and learning [358,359] Impair energy metabolism and contribute to fatigue and muscle weakness [360] Increased susceptibility to infections and inflammatory diseases [357,361]	Serotonin toxicity [363,364,365] Excessive production of kynurenine and its derivatives may contribute to oxidative stress and inflammation [366,367]
Tyrosine semi-essential aromatic	Biosynthesis of catecholamines [368,369] Precursor to thyroid hormones and melanin [370,371] Tyrosine phosphorylation, nitrosation, and sulfonation are critical PTMs that play pivotal roles in cellular signaling and regulation [372,373]	Tyrosine hydroxylase (TH) deficiency [374,375] Depression and anxiety [375,377]	Heightened physiological arousal and anxiety symptoms [378,379] Obesity and insulin resistance [380,381] Tyrosinemia type I—severe liver dysfunction, neurological crises, and rickets due to the accumulation of toxic metabolites [382] Tyrosinemia type II—skin lesions, corneal deposits, and neurological impairments [383,384] Tyrosinemia type III—neurological symptoms and intellectual disability [385,386]
Valine essential branched-chain	Muscle maintenance and repair [184] Stimulating mitochondrial function in muscle cells [387,388] Enhances insulin sensitivity [389,390] Influence on triglyceride synthesis and overall lipid profiles [388,391] Enhances intestinal health and immune status [392,393] Regulation of synthesis of neurotransmitter synthesis [394]	Loss in weight due to decreased protein synthesis and stimulated protein and fat break down [193,395] Increased insulin sensitivity [213,389] Neurotoxic effects [396,397]	Impaired insulin signaling and glucose metabolism [398,399] Cardiovascular complications [398,400] Impaired lipid metabolism and enhanced NAFLD [401,402]
